# A Novel *de Novo* 20q13.11q13.12 Microdeletion in a Boy with Neurodevelopmental Disorders − Case Report

**DOI:** 10.34763/devperiodmed.20172102.9194

**Published:** 2017-08-11

**Authors:** Joanna Bernaciak, Barbara Wiśniowiecka-Kowalnik, Jennifer Castañeda, Anna Kutkowska-Kaźmierczak, Beata Nowakowska

**Affiliations:** 1 Department of Medical Genetics Institute of Mother and Child, Warsaw, Poland

**Keywords:** aCGH, microdeletion 20q13.11q13.12, neurodevelopmental disorders, PTPRT gene, aCGH, mikrodelecja 20q13.11q13.12, zaburzenia neurorozwojowe, gen PTPRT

## Abstract

Copy-number variants (CNVs) are an important cause of human neurodevelopmental disorders. We present the first case of a 424 kb de novo 20q13.11q13.12 microdeletion in a patient with attention deficit disorder, tics and autistic behaviors, such as emotional and behavioral problems, and movement stereotypes. This region includes three genes expressed in the brain: SFRS6, PTPRT and L3MBTL. Our results suggest that loss of the chromosomal region 20q13.11q13.12 is causative for the clinical findings observed in the patient.

## Streszczenie

Zmiany liczby kopii fragmentów DNA (CNVs, ang. copy-number variants) są ważnym czynnikiem w patogenezie zaburzeń neurorozwojowych. Przedstawiamy pierwszy przypadek mikrodelecji w regionie 20q13.11q13.12 o wielkości 424 kpz powstałej de novo u pacjenta z takimi objawami klinicznymi jak: zaburzona koncentracja uwagi, tiki, zachowania autystyczne (problemy emocjonalne i zachowawcze) oraz stereotypie ruchowe. Aberracja obejmuje region zawierający trzy geny, które ulegają ekspresji w mózgu: SFRS6, PTPRT i L3MBTL. Wyniki naszych badań sugerują, że delecja regionu 20q13.11q13.12 jest odpowiedzialna za objawy kliniczne występujące u pacjenta.

## Introduction

Neurodevelopmental disorders are a group involving developmental impairments of the central nervous system. These include intellectual disability (ID), autism spectrum disorders (ASDs), attention deficit hyperactivity disorder (ADHD), impaired motor function, learning, language or non-verbal communication and tics [1]. Epidemiological studies have identified various risk factors for neurodevelopmental disorders. Submicroscopic chromosomal copy-number variants (CNVs) are some of the contributing factors. Because of the large genetic heterogeneity of neurodevelopmental disorders, high-resolution whole-genome analyses, such as array comparative genomic hybridization (aCGH), are useful tools to study the etiopathogenesis of these disorders and therefore aCGH is recommended as a first-tier clinical diagnostic test for patients with NI and ASDs [2]. Moreover, the advent of aCGH technologies has paved the way to the discovery of novel genes. We have identified and characterized a novel microdeletion in the 20q13.11q13.12 region in a boy with emotional and behavioral problems, movement stereotypes, tics and attention deficit disorder; we further report on associated clinical and neurological findings in the patient.

## Material and molecular investigation

The patient was born to non-consanguineous parents at 40 Hbd weeks of gestation by caesarean section. Body parameters were within the normal range with birth weight of 3250 g, body length 58 cm and head circumference 34 cm. Early developmental milestones were normally attained. At school age (from age 6) he was noted to have stereotyped movements, nervous tics, social and emotional disturbances, and attention deficit disorder. Currently – at age 13 − he still manifests nervous tics in stressful situations. He has been diagnosed with dyslexia, dysgraphia, attention deficit disorder and continues to have learning difficulties. His intelligence is assessed at average level. No significant dysmorphic characteristics have been observed aside from a slightly asymmetric and narrow chest, small ears, and synophrys. EEG analysis showed discrete changes on the right temporal area. The family history is positive for Asperger syndrome in his father’s brother, depression in his mother and schizophrenia in his paternal grandmother.

Oligonucleotide array comparative genomic hybridization (aCGH) was performed using 8x60K from Oxford Gene Technology (CytoSure ISCA v2). Array CGH has genome-wide coverage with an average resolution of 120 kb. DNA digestion, labeling, and hybridization were performed following the manufacturer’s instructions.

## Results

**Fig. 1:**
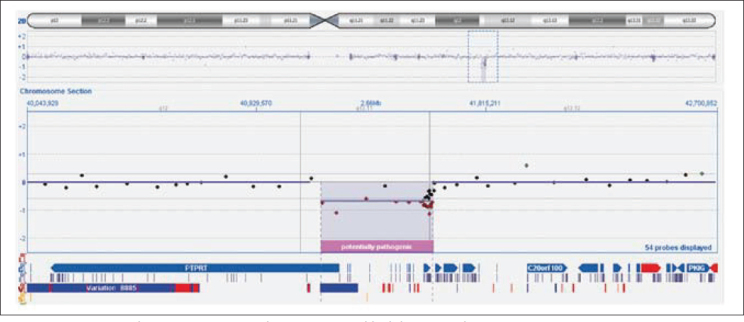
Array CGH analysis in our patient showing an 424 kb deletion in chromosome 20q13.11q13.12. Two genes are deleted: *SFRS6* , *L3MBTL* and first exon of *PTPRT* gene. Ryc. 1. W badaniu metodą aCGH u naszego pacjenta stwierdzono delecję regionu 20q13.11q13.12 o wielkości 424 kb. Delecji uległy dwa geny: SFRS6 , L3MBTL oraz pierwszy ekson genu PTPRT.

The interstitial deletion of 424 kb was identified at chromosome 20q13.11q13.12 using aCGH (43,117,849-43,541,875 - UCSC Genome Browser on Human Dec 2013 GRCh38/hg38). The deleted region contained three genes: *SFRS6* (*serine/arginine-rich splicing factor 6*, OMIM 601944), *L3MBTL* (*l3mbt, drosophila, homolog of, 1*, OMIM 608802) and the first exon of the *PTPRT* gene (*protein tyrosine phosphatase, receptor type, T*, OMIM 608712). Both parents were tested by aCGH and were negative for the aberration, indicating that the 20q13.11q13.12 microdeletion occurred *de novo* in the patient (fig. 1).

## Discussion

We performed a literature review and database search for individuals carrying a microdeletion involving the long arm of chromosome 20. To date, there are very few medical reports of 20q13 deletion, most of which involve regions more distal to our case [3, 4, 5]. We present, to our knowledge, the first case of 20q13.11q13.12 microdeletion encompassing the *PTRP* gene in a patient with neurodevelopmental disorder.

The deleted region observed in our patient includes three genes: *PTPRT, SFRS6* and *L3MBTL* expressed in many tissues, mostly in the brain. Two of these genes (*PTPRT* and *L3MBTL*) are haploinsuffcienct (sensitive to haploinsuffciency). The *PTPRT* gene is the candidate gene in the 20q13.11q13.12 region most likely associated with the pathology identified in our patient. Receptor protein tyrosine phosphatase (PTPRT) is a transmembrane protein that interacts with other cell adhesion molecules and regulates a variety of cellular processes including cell growth, differentiation, mitotic cycle, and oncogenic transformation. The PTPRT protein is a regulator of synaptic formation and neuronal development [6]. Overexpression of *PTPRT* in hippocampal neurons correlates with synaptic formation and dendritic arborization. On the other hand, knockdown of *PTPRT* decreased neuronal transmissions and attenuated neuronal development [6]. *PTPRT* was identified as a potential candidate gene for autism spectrum disorders (ASDs). A genome-wide association study found suggestive linkage results between the chromosome region 20q11.21 and ASDs [7]. One of the autism candidate genes of interest in this region was reported to be *PTPRT*. Further, Christian *et al*. identified an inherited duplication including this gene in a patient with ASDs [8].

A large group of the genes identified as risk factors for ASD plays an important role in synaptic homeostasis [9]. This indicates that disturbances of synaptic homeostasis are likely associated with ASD pathogenesis. PTPRT is a candidate protein for modulating proteins that play an important role in synaptic transmission and synapse formation. Rajamani et al. generated a knocking mouse line model of the *PTPRT* gene. They observed only a slight change in locomotor activities and anxiety-related behaviors in the homozygous knocking mice with *PTPRT* mutation, and higher social approach scores in male homozygous mice. It seems that PTPRT phosphatase function affects mouse social behaviors which are similar to ASDs patients’ symptoms [10]. Moreover, Schuurs-Hoeijmakers et al. identified potentially pathogenic mutations in five genes, among them in *PTPRT*, in siblings with intellectual disability [11]. Further, Licis *et al*. found 7 contiguous single nucleotide polymorphisms at chromosome 20q12q13.12 in all the affected individuals [9] from a single large family with manifestations of sleepwalking [12]. Our patient was diagnosed with attention deficit disorder and has stereotype movements, nervous tics, social and emotional disturbances. The above reports on gene, animal and clinical phenotype studies support our hypothesis that the *PTPRT* is a candidate gene for neurodevelopmental disorders, such as ADHD and ASDs.

The second gene deleted in our patient is *SFRS6*. The protein encoded by this gene belongs to the splicing factor SR family and has been shown to bind with and modulate another member of the family − SFRS12. SFRS6 has been found mainly in the brain. There are only several publications about the *SRSF6* gene – it is proposed as a candidate gene for colorectal tumors [13] and is associated with an increased risk of nonobstructive azoospermia [14].

The *L3MBTL* gene is also mainly expressed in the brain. This gene encodes a member of the polycomb proteins’ family which causes chromatin condensation and repression of transcription [15]. This protein is associated with cancer of hematopoietic cells [16].

## Conclusion

In summary, the established function of the *PTPRT* gene suggests that haploinsufficiency of this gene may result in the clinical features observed in our patient: stereotype movements, nervous tics, social and emotional disturbances and attention-span deficits. Little is known about the *SFRS 12* and *L3MBTL* genes, but in view of the fact that these genes are expressed mainly in the brain, we suspect that their deletion may also contribute to the pathogenesis of the developmental disturbances in our patient.
